# Cross-sectional and prospective associations between active living environments and accelerometer-assessed physical activity in the EPIC-Norfolk cohort

**DOI:** 10.1016/j.healthplace.2020.102490

**Published:** 2021-01

**Authors:** Samantha Hajna, Soren Brage, Alice Dalton, Simon J. Griffin, Andy P. Jones, Kay-Tee Khaw, Robert Luben, Nicholas J. Wareham, Jenna Panter

**Affiliations:** a Medical Research Council (MRC) Epidemiology Unit, University of Cambridge, Cambridge, United Kingdom; b Norwich Medical School, University of East Anglia, Norwich, UK; c Department of Public Health and Primary Care, University of Cambridge, Cambridge, United Kingdom

**Keywords:** Active living environments, Physical activity, Older adults

## Abstract

The environments in which young and middle-aged adults live may influence their physical activity (PA) behaviours. These associations are less clear among older adults. We estimated cross-sectional and prospective associations of population density, junction density, and land use mix and perceived active living environments with accelerometer-assessed PA in a cohort of older adults. Adults living in more dense and mixed neighbourhoods had less optimal activity profiles at baseline and less optimal changes in activity. Better perceptions were associated with more overall PA at baseline. Interventions for older adults may wish to target individuals living in more dense and mixed neighbourhoods.

## Highlights

• Older adults living in more dense/mixed areas are more sedentary and less active.
• More dense/mixed areas are associated with less optimal changes in activity.
• Adults who perceive living in more activity-friendly accumulate more total activity.
• There was no evidence of associations between perceptions and changes in activity.

## 1 Introduction

Physical inactivity increases the risk of morbidity and premature mortality (Lear et al., 2017). Despite this, 27.5% of adults are insufficiently active (Guthold et al., 2018). Older adults (≥65 years) are particularly inactive with substantial increases in sedentary time (ST) and decreases in physical activity (PA) observed with increasing age (Guthold et al., 2018; Hajna et al., 2018). To reduce health care costs associated with the treatment of inactivity-related complications, older age represents an important intervention period (Fern, 2009). Many countries have implemented national recommendations to promote increases in PA, improve health, and reduce the economic burden associated with the treatment of physical inactivity-related complications (Das and Horton, 2016). There is no evidence, however, that these recommendations have led to population-level reductions in ST and increases in PA (Guthold et al., 2018; Das and Horton, 2016).

PA interventions are effective in the short to medium term in controlled settings, but achieving sustained population-level increases in PA has been challenging (Reis et al., 2016). The scaling up of interventions requires a thorough understanding of the determinants of PA and coordinated action in targeting these determinants across multiple sectors of society (e.g. education, healthcare, urban planning, public transport) (Reis et al., 2016). Much work has been done on identifying the individual-level determinants of PA (Condello et al., 2017), but many unanswered questions remain regarding the environmental determinants of PA. Identifying the environmental determinants of PA is of interest because even small changes to environments may have large population-level impacts (Franco et al., 2015).

Active living environments (ALEs) is a term that describes the collective characteristics of neighbourhoods that may influence PA behaviours (Herrmann et al., 2019). Three macro-scale features of ALEs that have been linked to higher levels of PA include higher population density, junction density, and land use mix (Herrmann et al., 2019). While our understanding of the role that these characteristics play in PA behaviour is growing, this literature has relied heavily on cross-sectional study designs (Smith et al., 2017; Kärmeniemi et al., 2018; Barnett et al., 2017) participant-reported measures of exposures and/or outcomes (Smith et al., 2017; Barnett et al., 2017), and the study of young and middle-aged populations (Smith et al., 2017). Furthermore, despite evidence that different activity intensities may each hold important benefits for health (Dempsey et al., 2020; Ekelund et al., 2019), no study has examined the associations between these ALE measures and device-measured changes in activity across the entire activity intensity spectrum. To address these gaps in knowledge, we aimed to estimate the cross-sectional and prospective associations of objectively-assessed ALEs with accelerometer-assessed sedentary time (ST), moderate-intensity PA (MVPA), light-intensity PA (LPA), and overall PA in a cohort of older adults. Our secondary aim was to explore associations between participant-reported ALEs and activity intensities in this population.

## 2 Methods

We used data collected as part of the European Prospective Investigation of Cancer (EPIC)-Norfolk Study (Day et al., 1999). Ethics approval for the EPIC-Norfolk Study was approved by the East of England - Cambridge East ethics committee. Participants provided informed consent prior to participating in the study. A total of 25,639 participants were recruited between 1993 and 1997 from 35 general practices and invited to attend a health check. Following this first assessment (Health Check 1), participants were invited to attend 2nd (1998–2000), 3rd (2004–2011), and 4th (2012–2016) health checks. For the present study, we used data collected at the 3rd and 4th Health Checks (referred to herein as the baseline and follow-up visits, respectively) as these were the only health checks at which PA was assessed using accelerometers.

### 2.1 Active living environments

#### 2.1.1 Objectively-assessed

We calculated an objective ALE score for each study participant using Geographic Information System software (ArcMap 10.4.1; ESRI; Redlands, CA, USA). The score captured the population density, junction density, and land use mix of each participant's home neighbourhood. The data sources and the methods used to calculate the components of the score are provided in Supplemental Table 1. In brief, population density was defined as the number of residents in a postcode (zip-code) area/km^2^. Junction density and land use mix were derived for each participant's home neighbourhood (defined as 800-m polygonal street network buffers drawn around the centroid of the home postcode address). Junction density was defined as the number of junctions per neighbourhood area (hectares). Land use mix was calculated using the Herfindahl-Hirschman Index (HHI) (Rhoades, 1993). The HHI captured the degree of heterogeneity in thirteen major land uses (0: most mixed; 10,000: least mixed). The objective ALE score was calculated by summing the z scores of the population density, junction density, and land use mix variables with equal weight given to each variable. A higher objective ALE score was indicative of denser and more mixed neighbourhoods. All components of the ALE score were equally weighted.

#### 2.1.2 Participant-reported

We assessed participants' perceptions of the walking-friendliness of their home neighbourhoods using a 24-item modified version of the Neighbourhood Environment Walkability Survey (Supplemental Table 2). (Cerin et al., 2006) A composite score was calculated by summing the participants’ responses to these 24 items. A higher score was indicative of neighbourhoods that were perceived as more activity-friendly.

### 2.2 Physical activity

Participants wore accelerometers (ActiGraph, Pensacola, FL) on their right hips for seven days (except when bathing, swimming, and sleeping) immediately following the baseline and follow-up visits. Accelerometers were returned after seven days using a postage paid envelope. Uniaxial accelerometers (GT1M; data recorded in 5-s epochs) were worn at baseline. Triaxial accelerometers (GT3X+; data recorded at 100 Hz) were worn at follow-up. We harmonised the data collected using previously described methods (Hajna et al., 2018). We included participants who had ≥4 valid days of data (weekend or weekday) at each visit. A valid day was defined as ≥600 min/day of wear time and non-wear time was defined as time segments with ≥90 min of continuous zero activity counts. We excluded participants in whom data were not recorded in 5-s epochs and participants who wore their accelerometers ≥19 h/day (indicative of sleep wear). Our exposures of interest included ST, LPA, MVPA, and overall PA. ST, LPA, and MVPA were expressed in minutes/day using the following cut-offs: ST (<100 counts per minute; cpm), LPA (100–808 cpm), and MVPA (≥809 cpm), cut-points used previously in the EPIC-Norfolk cohort (Hajna et al., 2018; Berkemeyer et al., 2016). Overall PA was defined as total activity counts divided by valid wear time.

### 2.3 Covariates

A range of socio-demographic, geographical and health characteristics were assessed at the first EPIC-Norfolk assessment (1993–1997), at baseline, or at follow-up by trained research assistants or as part of the EPIC-Norfolk questionnaires (Table 1).

**Table 1:** Characteristics of the study population (n = 942).

Covariates	
	mean (SD)
Age, *years* (Range: 49–91 years)	67.6 (6.8)
Body mass index, *kg/m*^*2*^	26.3 (4.2)
	**% (n)**
Women	58.0 (546)
Education level^a^	
*O-level or lower*	33.9 (319)
*A-level*	46.9 (442)
*Degree*	19.2 (181)
Paid job at present	28.7 (270)
Married/living with partner (*vs.* single/widowed/separated/divorced)	84.9 (800)
Employment-based social class	
*Unskilled/semi-skilled*	10.5 (99)
*Skilled (non-manual and manual)*	36.5 (344)
*Professional/managerial*	53.0 (499)
Born in the UK	97.5 (918)
Smoking status (current *vs.* former/never)	2.7 (25)
Physical disability that limits walking	9.6 (90)
Car primary mode of transport outside of work^b^	90.8 (855)
Dog ownership	18.9 (178)
Self-rated health (good/very good/excellent self-rated health *vs.* fair/poor)	89.2 (840)
Urban home neighbourhood (*vs.* rural)	53.3 (502)
**Environment measures**	
	**mean (SD)**
Objective ALE score^c^	0 (2.4)
Population density, *residents/km*^*2*^	2,069.3 (1715.8)
Junction density,^e^*junctions/hectare*	22.3 (11.7)
Land use mix,^f^*HHI (0 = most mixed; 10,000 = least mixed)*	2,980.3 (1163.5)
Perceived ALE score	67.8 (9.3)
**Activity measures**	
	**mean (SD)**
Activity at baseline	
ST, *min/day*	667.0 (63.3)
LPA, *min/day*	107.5 (26.9)
MVPA, *min/day*	93.7 (36.9)
Overall PA, *cpm*	278.0 (116.5)
Changes in activity	
ST, *min/day/year*	3.1 (12.6)
LPA, *min/day/year*	−1.6 (5.2)
MVPA, *min/day/year*	−3.1 (6.0)
Overall PA, *cpm/year*	−8.9 (19.2)

Note: SD, standard deviation; ALE, active living environment; HHI, Herfindahl-Hirschman Index; cpm, counts per minute; All of the variables were assessed via a standardised questionnaire that was administered at baseline, with the exception of the changes in activity variables, education, employment-based social class, body mass index, and immigrant status. The changes in activity variables were assessed using accelerometer data collected at the baseline and follow-up visits. Education and employment-based social class were assessed as part of the 1993–1997 EPIC-Norfolk questionnaire. Place of birth used to assess immigrant status was assessed in a questionnaire that was mailed to participants between Health Check 2 (1998–2000) and Health Check 3 (2004–2011). Body mass index (kg/m^2^) was based on height and weight measurements collected by trained research assistants at the baseline and follow-up visits.

a The respective categories are approximately analogous to high school, college, and university.

b Compared to walking, using public transport, or cycling.

c The mean is equal to 0 as the score represents the sum of z-scores of population density, junction density, and land use mix.

### 2.4 Statistical analyses

Participants were included in the present analyses if they had complete information on exposures, outcomes, and covariates and lived at the same address at baseline and follow-up. All analyses were conducted in Stata/SE 14.1 (College Station, TX: StataCorp LP) and descriptive statistics were produced for all variables of interest. We used multivariate linear regression models to estimate mean differences in activity across quartiles of the ALE measures and the individual components of the ALE score to determine if any component of the score was particularly important. In the cross-sectional analyses, mean differences in activity were presented in their original units. In the prospective analyses, mean differences in changes in activity were expressed as rates of change normalised for follow-up time to account for variations in follow-up time.

Our analyses were guided by a conceptual model developed *a priori* based on the existing ALE-activity literature. The sequential order in which covariates were added into the models are outlined in Supplemental Table 3. In brief, for the cross-sectional models, we adjusted for covariates as they were assessed at baseline (or between 1993 and 1997, if not assessed at baseline). For the prospective models, we also adjusted for those covariates from the cross-sectional models plus baseline activity and changes in employment status, marital status, season of assessment, and accelerometer wear-time. For both the cross-sectional and prospective perceived ALE-activity models, we adjusted for the objective ALE score. This was because we wanted to estimate how perceptions of ALEs were associated with activity independent of actual urban designs. We did not, however, adjust for the perceived ALE score in the objective ALE-activity models. This was because we hypothesised that perceptions lie on the causal pathway linking objectively-assessed ALEs to activity and we wanted to estimate the ALE-activity associations allowing for the potential mediating effect of perceptions.

## 3 Results

**Fig. 1:**
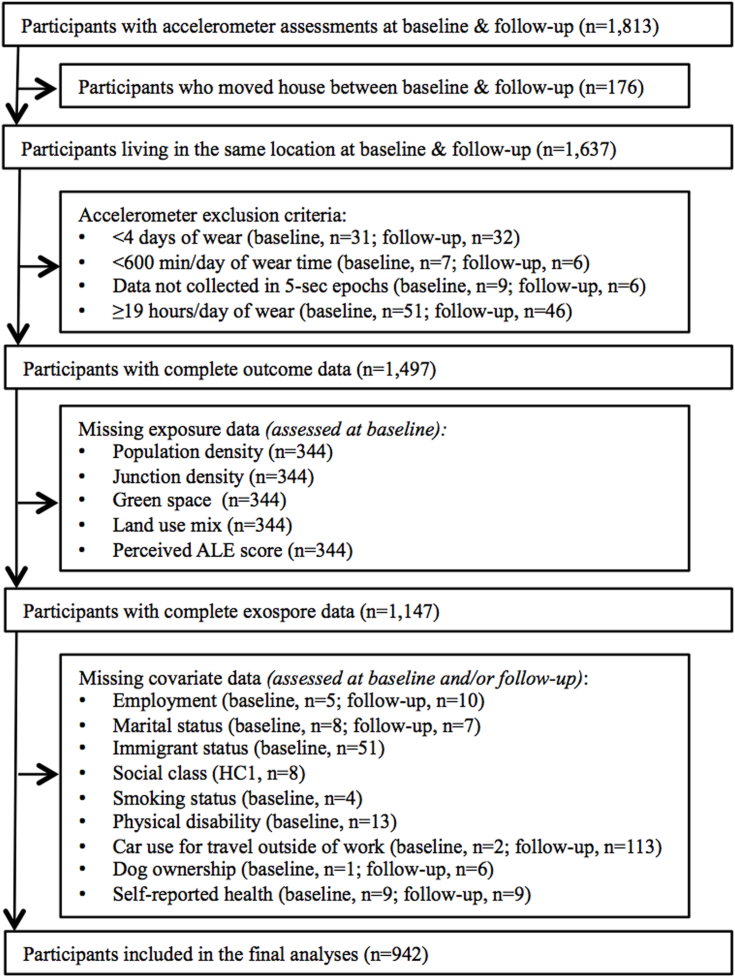
Selection of participants into the present study. Note: HC1, Health Check 1; ALE, active living environment; Since some participants had multiple applicable exclusion criteria, the sum of the individual excluded observations is not equivalent to the total number of excluded observations.

A total of 1,813 participants attended the baseline and follow-up visits and took part in the accelerometry assessment. Of these, 942 participants had not moved home between baseline and follow-up and had complete exposure, outcome, and covariate data (Fig. 1).

### 3.1 Descriptive characteristics

The characteristics of the study population are provided in Table 1. In brief, at baseline, participants were aged 67.6 years (SD = 6.8), the majority were women (58.0%), most were married/living with a partner (84.9%), and 28.7% had a paid job. Participants accumulated an average of 11.2 h/day of ST, 1.8 h/day of LPA, 1.6 h/day of MVPA, and 278.0 cpm of overall PA. Over the follow-up period (5.7 ± 2.0 years), ST increased by 15.4 min/day, and LPA, MVPA, and overall PA declined by 8.3 min/day, 16.5 min/day and 49.0 cpm, respectively. There were some differences between participants who were included in the study and those who were excluded due to having attended only the baseline visit (e.g. included participants were younger, more likely to have a degree, and more likely to have a job compared to excluded participants; Supplemental Table 4).

### 3.2 Cross-sectional associations

Compared to participants living in the least dense and least mixed neighbourhoods as assessed by the objective ALE score, participants living in the most dense and most mixed neighbourhoods (i.e. Q4 vs. Q1) accumulated 19.5 min/day more ST (95% CI 7.9, 31.1), 10.8 min/day less LPA (95% CI -16.5, −5.1), and 8.7 min/day less MVPA (95% CI -16.5, −0.8). The associations were similar for the individual components of the objective ALE score (Table 2). Participants who reported living in the most compared to the least activity-friendly (Q4 vs. Q1) accumulated 27.1 cpm more overall PA (95% CI 5.1, 49.1; Table 2). Associations were similar in 1) analyses treating the ALE measures as continuous scores (Supplemental Table 5), 2) unadjusted and less adjusted models (Supplemental Table 6), and 3) cross-sectional analyses in which we used the full baseline dataset (i.e. not excluding those who did not have complete follow-up data; Supplemental Table 7).

**Table 2:** Cross-sectional associations showing maximally-adjusted means differences (95% confidence intervals) in activity across quartiles of the ALE measures at baseline (n = 942).

	ST *(min/day)*	LPA (*min/day)*	MVPA (*min/day)*	Overall PA (*cpm)*
**Objective ALE score**				
Q1 (Least dense/mixed)	REF	REF	REF	REF
Q2	**16.8 (7.1, 26.5)**	**−7.1 (-11.9, -2.3)**	**−9.7 (-16.3, -3.2)**	**−25.7 (-46.5, -4.9)**
Q3	**18.6 (8.0, 29.1)**	**−8.3 (-13.5, -3.1)**	**−10.3 (-17.4, -3.2)**	**−29.3 (-51.9, -6.7)**
Q4 (Most dense/mixed)	**19.5 (7.9, 31.1)**	**−10.8 (-16.5, -5.1)**	**−8.7 (-16.5, -0.8)**	−16.6 (−41.4, 8.3)
**Population density**				
Q1 (Least dense)	REF	REF	REF	REF
Q2	**17.4 (7.9, 27.0)**	**−7.3 (-12.0, -2.6)**	**−10.2 (-16.6, -3.7)**	**−27.6 (-48.2, -7.1)**
Q3	**14.2 (2.9, 25.6)**	**−8.3 (-13.9, -2.8)**	−5.9 (−13.6, 1.8)	−14.0 (−38.3, 10.4)
Q4 (Most dense)	**22.0 (9.0, 35.1)**	**−11.5 (-17.9, -5.0)**	**−10.6 (-19.4, -1.7)**	−22.2 (−50.2, 5.8)
**Junction density**				
Q1 (Least dense)	REF	REF	REF	REF
Q2	**11.1 (1.8, 20.4)**	**−6.1 (-10.7, -1.6)**	−5.0 (−11.3, 1.3)	−14.3 (−34.2, 5.6)
Q3	**18.6 (8.3, 28.9)**	**−8.7 (-13.8, -3.6)**	**−9.9 (-16.9, -3.0)**	**−25.3 (-47.4, -3.2)**
Q4 (Most dense)	**14.4 (3.6, 25.1)**	**−9.2 (-14.5, -3.9)**	−5.2 (−12.4, 2.1)	−8.1 (−31.2, 15.0)
**Land use mix**				
Q1 (Least mixed)	REF	REF	REF	REF
Q2	6.9 (−2.0, 15.7)	−3.5 (−7.8, 0.9)	−3.4 (−9.4, 2.6)	−9.6 (−28.5, 9.4)
Q3	4.3 (−4.8, 13.4)	−2.4 (−6.9, 2.1)	−1.9 (−8.0, 4.3)	−1.0 (−20.5, 18.6)
Q4 (Most Mixed)	**15.7 (6.8, 24.5)**	**−8.1 (-12.4, -3.7)**	**−7.6 (-13.6, -1.6)**	**−20.2 (-39.3, -1.2)**
**Perceived ALE score**				
Q1 (Least activity-friendly)	REF	REF	REF	REF
Q2	1.7 (−7.4, 10.8)	−2.2 (−6.7, 2.2)	0.6 (−5.6, 6.7)	7.9 (−11.5, 27.3)
Q3	1.7 (−8.1, 11.4)	−2.6 (−7.4, 2.1)	1.0 (−5.6, 7.5)	7.1 (−13.8, 27.9)
Q4 (Most activity-friendly)	−4.3 (−14.6, 6.0)	−1.3 (−6.4, 3.7)	5.6 (−1.4, 12.6)	**27**.**1 (5**.**1, 49**.**1)**

Note: ALE, active living environment; ST, sedentary time; LPA, light-intensity physical activity; MVPA, moderate-to-vigorous intensity physical activity; PA, physical activity; cpm, counts per minute; REF, reference.

Bolded values represent statistically significant effect estimates. Estimates are adjusted for all of the variables included in Blocks 1–3.

Quartile cutoffs (n): Objective ALE score Q1: <-1.3 (236), Q2≥−1.3<0.3 (238), Q3≥0.3<1.6 (233), Q4≥1.6 (235); Population density, residents/km^2^ Q1: <513.2 (236), Q2: ≥513.2<2,048.0 (235), Q3: ≥2,048.0<3,227.0 (237), Q4: ≥3,227.0 (234); Junction density, junctions/hectare (n): Q1: <12.8 (236), Q2: ≥12.8<22.1 (235), Q3: ≥22.1<29.6 (237), Q4: ≥29.6 (234); Land use mix Q1: <6,467.6 (236), Q2: ≥6,467.6<7,284.7 (235), Q3: ≥7,284.7<7,847.6 (236); Q4: ≥7,847.6 (235); Perceived ALE score Q1: <62 (239), Q2: ≥62<68 (238), Q3: ≥68<74(240), Q4: ≥74 (225).

### 3.3 Prospective associations

**Fig. 2:**
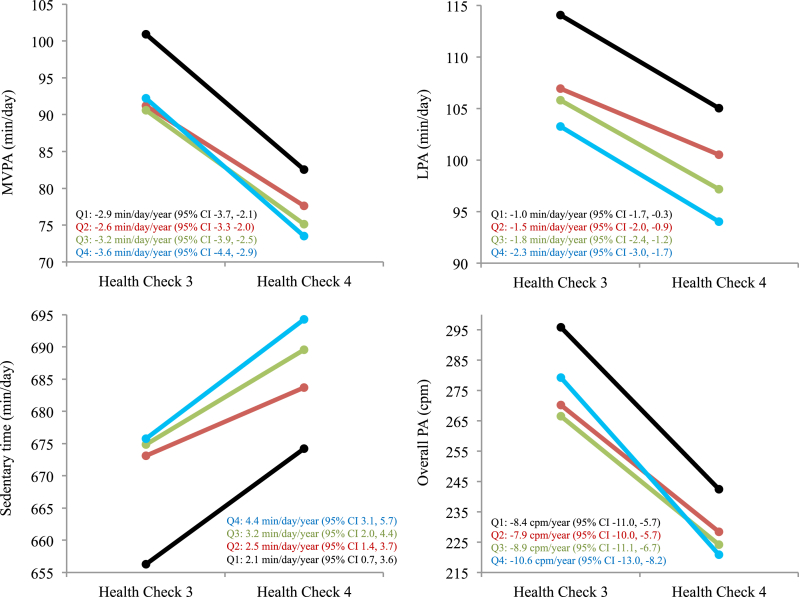
Maximally-adjusted mean activity at baseline and follow-up and corresponding rates of change across quartiles of the objective active living environment score. CI, confidence interval; Q, quartile; MVPA, moderate-to-vigorous intensity activity, LPA, light-intensity physical activity; PA, physical activity.

Increases in ST were 2.3 min/day/year greater (95% CI 0.1, 4.5) and declines in LPA were 1.4 min/day/year greater (95% CI -2.4, −0.3) among participants living in the most dense and most mixed neighbourhoods compared to the least dense and least mixed neighbourhoods as assessed by the objective ALE score (Q4 vs. Q1; Table 3) Corresponding maximally-adjusted rates of change across objective ALE score quartiles are presented in Fig. 2. The findings were generally consistent for the individual components of the objective ALE score and in analyses treating the ALE measures as continuous variables (Supplemental Table 5). Participants' perceptions of the ALEs were not associated with participants’ changes in any of the activity intensities. The maximally adjusted ALE-activity associations were generally similar to those observed in less adjusted models (Supplemental Table 8).

**Table 3:** Prospective associations showing maximally-adjusted mean differences (95% confidence intervals) in changes in activity across quartiles of the baseline ALE measures (n = 942).

	ST *(min/day/year)*	LPA *(min/day/year)*	MVPA (*min/day/year)*	Overall PA (*cpm/year)*
**Objective ALE score**				
Q1 (Least dense/mixed)	REF	REF	REF	REF
Q2	0.4 (−1.5, 2.2)	−0.5 (−1.4, 0.4)	0.3 (−0.7, 1.3)	0.5 (−2.9, 3.9)
Q3	1.0 (−1.0, 3.0)	−0.8 (−1.8, 0.2)	−0.3 (−1.4, 0.8)	−0.5 (−4.2, 3.2)
Q4 (Most dense/mixed)	**2.3 (0.1, 4.5)**	**−1.4 (-2.4, -0.3)**	−0.7 (−2.0, 0.5)	−2.2 (−6.3, 1.9)
**Population density**				
Q1 (Least dense)	REF	REF	REF	REF
Q2	0.9 (−0.9, 2.8)	−0.5 (−1.4, 0.4)	0.05 (−1.0, 1.1)	−0.5 (−3.9, 2.8)
Q3	1.7 (−0.5, 3.8)	−0.7 (−1.8, 0.3)	−0.05 (−1.3, 1.2)	0.3 (−3.7, 4.3)
Q4 (Most dense)	2.1 (−0.3, 4.6)	**−1.6 (-2.9, -0.4)**	−0.6 (−2.0, 0.8)	−1.5 (−6.0, 3.1)
**Junction density**				
Q1 (Least dense)	REF	REF	REF	REF
Q2	1.3 (−0.4, 3.1)	−0.8 (−1.7, 0.04)	−0.5 (−1.5, 0.5)	−1.7 (−5.0, 1.5)
Q3	1.3 (−0.6, 3.3)	**−1.2 (-2.2, -0.3)**	−0.4 (−1.5, 0.7)	−0.8 (−4.4, 2.9)
Q4 (Most dense)	**2.5 (0.5, 4.6)**	**−1.2 (-2.2, -0.2)**	−0.9 (−2.1 0.2)	−3.0 (−6.8, 0.8)
**Land use mix**				
Q1 (Least mixed)	REF	REF	REF	REF
Q2	**2.1 (0.4, 3.7)**	**−1.0 (-1.8, -0.2)**	−0.7 (−1.6, 0.3)	−1.5 (−4.6, 1.6)
Q3	1.2 (−0.5, 2.9)	−0.4 (−1.2, 0.5)	−0.8 (−1.8, 0.2)	**−3.2 (-6.3, -0.03)**
Q4 (Most Mixed)	1.2 (−0.5, 2.9)	**−1.1 (-1.9, -0.3)**	−0.5 (−1.5, 0.4)	−1.7 (−4.8, 1.4)
**Perceived ALE score**				
Q1 (Least activity-friendly)	REF	REF	REF	REF
Q2	0.4 (−1.3, 2.1)	−0.7 (−1.5, 0.1)	−0.3 (−1.3, 0.6)	−0.9 (−4.0, 2.3)
Q3	0.6 (−1.2, 2.5)	−0.2 (−1.0, 0.7)	−0.4 (−1.4, 0.6)	−0.1 (−3.5, 3.3)
Q4 (Most activity-friendly)	0.3 (−1.6, 2.3)	−0.4 (−1.3, 0.5)	−0.1 (−1.2, 1.0)	0.9 (−2.7, 4.5)

Note: ALE, active living environment; ST, sedentary time; LPA, light-intensity physical activity; MVPA, moderate-to-vigorous intensity physical activity; PA, physical activity; cpm, counts per minute; REF, reference.

Bolded values represent statistically significant effect estimates. Estimates are adjusted for all of the variables included in Blocks 1–3.

Quartile cutoffs (n): Objective

ALE score Q1: <-1.3 (236), Q2: ≥-1.3<0.3 (238), Q3: ≥0.3<1.6 (233), Q4: ≥1.6 (235); Population density, residents/km^2^ Q1: <513.2 (236), Q2: ≥513.2<2,048.0 (235), Q3: ≥2,048.0<3,227.0 (237), Q4: ≥3,227.0 (234); Junction density, junctions/hectare Q1: <12.8 (236), Q2: ≥12.8<22.1 (235), Q3: ≥22.1<29.6 (237), Q4: ≥29.6 (234); Land use mix Q1: <6,467.6 (236), Q2: ≥6,467.6<7,284.7 (235), Q3: ≥7,284.7<7,847.6 (236); Q4: ≥7,847.6 (235); Perceived ALE score Q1: <62 (239), Q2: ≥62<68 (238), Q3: ≥68<74 (240), Q4: ≥74 (225).

## 4 Discussion

We found that participants living in more dense and mixed neighbourhoods based on large-scale objectively-assessed ALE measures had less optimal activity profiles at baseline and less optimal changes in activity over follow-up. Specially, participants living in the most compared to the least dense and mixed neighbourhoods accumulated 19.5 min/day more ST, 10.8 min/day less LPA, and 8.7 min/day less MVPA. Increases in ST and declines in LPA were greater in participants living in the most compared to the least dense and mixed neighbourhoods (+2.3 min/day/year and −1.4 min/day/year, respectively). Participants who reported living in the most compared to the least activity-friendly neighbourhoods accumulated 27.1 counts/minute more overall PA. Perceived ALEs were not associated with changes in ST or PA.

Evidence from cross-sectional studies linking objectively-assessed ALEs to device-measured activity is mixed with some studies reporting positive associations and others reporting inverse, null, or weak associations (Hajna et al., 2015a; Ferdinand et al., 2012; Oakes et al., 2007). For example, in a study of adults (18–66 years) from 14 cities in 10 countries across five continents, a 1,000 dwellings/km^2^ higher population density was associated with 0.6% more min/day of MVPA (95% CI 1.003 to 1.009) and a 100 intersections/km^2^ higher junction density was associated with 6.9% more min/day of MPVA (95% CI 1.011 to 1.130), but land use mix was not associated with MVPA (Sallis et al., 2016). In contrast, in a study of adults (24–86 years) in which a matched sampling design was used, neither population density nor junction density were associated with accelerometer-assessed activity (Oakes et al., 2007). Similarly, in a recent study of older Taiwanese adults (>60 years), population density, street connectivity, access to destinations, and public transportation were not associated with accelerometer-assessed activity (Chen et al., 2019). Several possible explanations for the divergent results across cross-sectional studies for macro-scale measures of density and mix include that these associations vary by context (e.g. geography and culture) and age group, differences in the heterogeneity of the ALE measures of interest across study populations, measurement error, model misspecification, reliance on statistical testing rather than the interpretation of findings in the context of the variance estimates, and/or inadequate control of confounding (Oakes et al., 2007; Chen et al., 2019; Forsyth et al., 2007; Van Cauwenberg et al., 2011).

There is some evidence that environmental changes are associated with PA (Giles-Corti et al., 2013; Wasfi et al., 2016), but other studies have shown null associations. For example, accelerometer-assessed daily steps and MVPA did not increase over two years in adults who moved to East Village, the former London 2012 Olympic and Paralympic Games Athletes’ Village that was designed to have a high level of access to public transport, cycle paths, greenspace, street furniture, and a variety of community facilities and retail outlets (Nightingale et al., 2019). There was also no evidence of differences in changes in self-reported walking over three years among adults living in Australia who moved to neighbourhoods that were more or less activity-friendly based on urban design features (Christian et al., 2013). Recent systematic reviews on the longitudinal associations between built environments and PA suggest that although there is some evidence that changes in built environments may be linked to PA (Kärmeniemi et al., 2018; Ding et al., 2018), the evidence is weaker in methodologically stronger prospective longitudinal relocation studies (Ding et al., 2018) and for some objectively-assessed measures of environments (e.g. street connectivity) (Kärmeniemi et al., 2018). Our study adds to the literature by demonstrating that for older adults neither living in more dense and more mixed neighbourhoods nor perceiving neighbourhoods to be more activity-friendly was associated with beneficial changes in activity.

Our study is the first to estimate the cross-sectional and prospective associations of objectively-assessed population density, junction density and land use mix and perceptions of ALEs with accelerometer-assessed ST, LPA, MVPA, and overall PA among older adults. Our finding that more mixed/dense neighbourhoods are associated with less optimal activity profiles among older adults is consistent with evidence that the correlates of PA differ across age subgroups (Plotnikoff et al., 2004), and that denser and more mixed ALEs are associated with lower diabetes incidence in adults <65 years but not among adults ≥65 years (Booth et al., 2019). While living in neighbourhoods that are denser and more mixed may facilitate PA among young and middle-aged adults (Smith et al., 2017), our study suggests that older adults may not be disadvantaged by living less dense and less mixed-use neighbourhoods. This may be because less mixed/dense neighbourhoods could facilitate outdoor activities that older adults enjoy doing (e.g. walking, outdoor recreation, or gardening (Szanton et al., 2015) by providing more outdoor space and quieter/less busy environments while walking for transport may be most supported by the classic “walkable” environment and may become less frequent in older age groups. Our finding that participants who reported better ALEs accumulated higher levels of accelerometer-assessed activity at baseline was also consistent with previous findings (Strath et al., 2012; Van Dyck et al., 2015). Whilst better perceptions of neighbourhood environments may lead to higher levels of activity, it is also possible that being more active may improve perceptions of neighbourhood environments. Given that the investigation of the perceived ALE-PA associations was only exploratory in our study, the associations between individual components of the perceived ALE score and changes in PA could be investigated in future studies of older adults to identify which specific perceptions are particularly associated with activity over the longer-term in this population.

The differences in activity that we observed between ALE quartiles (e.g. an approximately 9 min/day higher MVPA adults living in the most compared to the least mixed/dense neighbourhoods) were potentially clinically important. In a prospective population-based cohort study of older men recruited from general practices in the UK, it was found that every additional 10 min/day in MVPA was associated with a 10% decreases risk of all-cause mortality over the median five-year follow-up period (Jefferis et al., 2018). Studies on ST and LPA have generally quantified morbidity and mortality risk for 30 min/day increases in ST and LPA (Jefferis et al., 2018; Qiu et al., 2020) or across quartiles of overall PA, ST and LPA (Ekelund et al., 2019; Lee et al., 2018; Tarp et al., 2020). Because of this, direct comparisons to the differences we observed in our study are difficult. Nevertheless, given evidence that there is a dose-response relationship between these activity intensities and risk of premature mortality (Dempsey et al., 2020; Ekelund et al., 2019), smaller difference in ST, LPA, and overall PA such as those observed in our study may be clinically important.

Although more studies that examine the cross-sectional and prospective associations of objectively-assessed ALEs and device-measured PA are required among older adults to corroborate our findings, the results of our study suggest that interventions aimed at improving activity profiles of older adults, in both short and longer-terms, should target individuals living in denser and more mixed neighbourhoods. To inform the development of these interventions future research should investigate which components of less dense and mixed neighbourhoods help facilitate activity levels in activity in older adults. For example, there is emerging evidence that green spaces are associated with decreased risk of premature mortality (Rojas-Rueda et al., 2019). Understanding how specific attributes of neighbourhoods (e.g. access to greenspaces) facilitate health behaviours in older adults will be critical in informing public policies that will help facilitate the creation of environments that support active lifestyles for older adults and mitigate their chronic disease risk.

Strengths of our study include the measurement of neighbourhoods using both perceived and objective measures, the device-measured assessment of PA, a large and relatively compliant and stable sample of older adults, a prospective study design, a relatively long-follow up period, the control of individual-level rather than area-level covariates, and the assessment of ALE-activity associations across the entire activity intensity spectrum. Several limitations should also be noted. First, the subset of EPIC-Norfolk participants that were included in our analyses had more optimal sociodemographic profiles than excluded participants. The generalizability of our findings may therefore be limited due to a healthy cohort bias (Day et al., 1999). Second, course measures of neighbourhood environments (e.g. population density) may miss finer-scaled environmental features that are important for PA. This may in part explain why we found that perceptions, a measure that captures participants' views on finer-scaled features, were associated with PA. Third, since postcodes are proxies for participants' addresses, the derived ALE measures are approximations of the participants’ actual active living environments. Fourth, since a large number of associations were assessed, some of the statistically significant results may have arisen by chance. Fifth, our study linked measures of ALE to activity that did not necessarily occur in those environments. It is possible that ALEs might only be linked to activity that occurs in there. Sixth, we did not control for potential confounding by resident self-selection (Cao et al., 2009). Lastly, there are some inherent limitations to our objective ALE measures (e.g. modifiable areal unit problem (Tribby et al., 2016). Given evidence that varying buffer shapes and sizes do not importantly alter associations between large-scale ALE measures and activity (Hajna et al., 2015b), we suspect that this is unlikely to significantly bias our findings. Future researchers may, however, wish to replicate our findings using other exposure assessment methods (e.g. activity space-based measures (Tribby et al., 2016).

In conclusion, our study suggests that older adults living in more dense and more mixed neighbourhoods have less optimal activity profiles than older adults living in less dense and less mixed neighbourhoods. Older adults who do not live in less dense and less mixed use neighbourhoods may not be disadvantaged in terms of their ability to engage in PA. We also found that older adults perceptions of their neighbourhoods may be associated with overall levels of activity cross-sectionally, but not with changes in activity. Interventions aimed at increasing activity in older adults may wish to target individuals living in more dense and mixed neighbourhoods.

## Data sharing

Researchers wishing to access the EPIC-Norfolk data should consult the MRC Epidemiology Unit meta-data access portal (https://epi-meta.mrc-epid.cam.ac.uk/).

## Declaration of competing interest

The authors declare they have no actual or potential competing financial interests.
