# Supplementary material for: Cross-sectional and prospective associations between active living environments and accelerometer-assessed physical activity in the EPIC-Norfolk cohort
Source: Health Place. 2021 Jan;67:102490. doi: 10.1016/j.healthplace.2020.102490 (PMC7883217; doi:10.1016/j.healthplace.2020.102490)
Supplement: Multimedia component 2 [file mmc2.docx]

| **Supplemental Table 1.** Description and data sources of the components of the objectively-assessed ALE score. | | |
| --- | --- | --- |
|  | **Description** | **Data Source(s)** |
| Population density | Number of residents in a postcode area/km^2^ of that postcode area. | Office for National Statistics, 2001 Census |
| Junction density | Number of nodes per neighbourhood area. | OS MasterMap® 2008 Integrated Transport Network Layer |
| Land use mix | Land use mix was calculated using the Herfindahl-Hirschman Index (HHI).  The HHI captured the heterogeneity (0: most mixed; 10,000: least mixed) in thirteen major land uses (public access land, sandy beaches, saltmarsh, sea/estuary, residential, gardens, commercial/industrial, other urban, woodland, farmland, grassland, uncultivated land, and small settlement) in each participants neighbourhood.  *HHI =* Σk*(*p_k_*100)^2  p = the proportion of land area devoted to the specific land use (k) in each buffer.  p_k_ = the land area devoted to a specific land use divided by the total area of walkable land uses in each buffer.  *Note:* HHI=10,000 (i.e. no mix) if none or only one of the relevant land uses fell into the buffer. We reverse coded the HHI prior to calculating the objective ALE score so that a higher HHI was indicative of greater heterogeneity. | Land Cover Map 2000, Centre for Ecology & Hydrology Land Cover*^®^* |

^a^ALE: active living environment.

| **Supplemental Table 2.** The 24-items included in the modified version of the Neighborhood Environment Walkability Scale^a^ and that were used in the calculation of the composite Perceived ALE score. | |
| --- | --- |
| For the following statements, please circle the answer that best applies to you and your neighbourhood. Please circle one answer per statement. In the statements, *within easy walking distance* means within a 10-15 minute walk from your home. | |
| **1** | There are shops to visit within easy walking distance of my home. |
| **2** | There is a park or open space to visit within easy walking distance of my home. |
| **3** | There is a sports or leisure centre within easy walking distance of my home. |
| **4** | It is pleasant to walk in my neighbourhood. |
| **5** | There are pedestrian crossings to help walkers cross busy streets in my neighbourhood. |
| **6** | I feel generally safe walking in my neighbourhood. |
| **7** | The crime rate in my neighbourhood makes it unsafe to go on walks at night. |
| **8** | It is easy to walk to a bus stop from my home. |
| **9** | There are few cul-de-sacs (dead-end streets) in my neighbourhood. |
| **10** | There are a lot of busy junctions in my neighbourhood. |
| **11** | There are major barriers to walking in my neighbourhood that make it hard to get from place to place (for example, busy roads, railway lines, rivers, hills). |
| **12** | There are many alternative routes for getting from place to place in my neighbourhood (I don’t have to go the same way every time). |
| **13** | There are pavements on most of the streets in my neighbourhood. |
| **14** | There are cycle paths in or near my neighbourhood that are easy to get to. |
| **15** | There is a verge that separates the streets from the pavements in my neighbourhood. |
| **16** | There are trees along the streets in my neighbourhood. |
| **17** | There are diverse and interesting things to look at in my neighbourhood (e.g. buildings and views). |
| **18** | There is so much traffic along nearby streets that it makes it difficult or unpleasant to walk in my neighbourhood. |
| **19** | There is so much traffic along nearby streets that it makes it difficult or unpleasant to cycle in my neighbourhood. |
| **20** | The speed of traffic on most nearby streets is usually slow (30 mph or less). |
| **21** | Most drivers exceed the posted speed limits while driving in my neighbourhood. |
| **22** | My neighbourhood streets are well lit at night. |
| **23** | Walkers and cyclists on the streets in my neighbourhood can be easily seen by people in their homes. |
| **24** | There is a high crime rate in my neighbourhood. |

Note: ALE, active living environment; Response categories included: strongly disagree (1), somewhat disagree (2), somewhat agree (3), and strongly agree (4).

^a^ Cerin E, Saelens BE, Sallis JF, Frank LD. Neighborhood environment walkability scale: Validity and development of a short form. *Med Sci Sports Exerc.* 2006.

| **Supplemental Table 3.** Summary of the covariates included in each of the models. | | | | | | | | | |
| --- | --- | --- | --- | --- | --- | --- | --- | --- | --- |
|  |  |  | **Model A** |  | **Model B** |  | **Model C** |  | **Model D** |
| **BLOCK 1**  Confounders | Age |  |  |  | ✔ |  | ✔ |  | ✔ |
|  | Education |  |  |  | ✔ |  | ✔ |  | ✔ |
|  | Employment ^a^ |  |  |  | ✔ |  | ✔ |  | ✔ |
|  | Marital status ^a^ |  |  |  | ✔ |  | ✔ |  | ✔ |
|  | Immigrant status |  |  |  | ✔ |  | ✔ |  | ✔ |
|  | Social class |  |  |  | ✔ |  | ✔ |  | ✔ |
|  | Urban status |  |  |  | ✔ |  | ✔ |  | ✔ |
|  |  |  |  |  |  |  | **+** |  | **+** |
|  |  |  |  |  |  |  |  |  |  |
| **BLOCK 2**  Predictors of activity outcomes | Sex |  |  |  |  |  | ✔ |  | ✔ |
|  | Smoking status |  |  |  |  |  | ✔ |  | ✔ |
|  | Season of assessment ^a,b^ |  |  |  |  |  | ✔ |  | ✔ |
|  | Accelerometer wear-time ^a,c^ |  |  |  |  |  | ✔ |  | ✔ |
|  | Physical disability |  |  |  |  |  | ✔ |  | ✔ |
|  | *Baseline activity ^d^* |  |  |  |  |  | ✔ |  | ✔ |
|  |  |  |  |  |  |  |  |  | **+** |
| **BLOCK 3**  Confounders and/or mediators | BMI ^a^ |  |  |  |  |  |  |  | ✔ |
|  | Car use outside of work ^a^ |  |  |  |  |  |  |  | ✔ |
|  | Dog ownership ^a^ |  |  |  |  |  |  |  | ✔ |
|  | Self-rated health ^a^ |  |  |  |  |  |  |  | ✔ |
|  | *Objective ALE score ^e^* |  |  |  |  |  |  |  | ✔ |

Note: ALE: active living environment

^a^ Covariates that could change differentially over the follow-up period and on which we had data at the baseline and follow-up visits. Changes in BMI and accelerometer wear-time were expressed as delta change from baseline to follow-up. Changes in the other variables were accounted for in the models via indicator variables (i.e. 0=no change; 1=a change from one category to another).

^b^ Seasons of assessment were based on the start date of the accelerometer wear periods at the baseline and follow-up visits and were coded as a continuous periodic variable, where spring = sin (2*π*day of year/365.25) and winter = cos (2*π*day of year/365.25)).

^c^ Accelerometer wear-time was based on the accelerometer data collected at the baseline and follow-up visits.

^d^ Baseline activity was only adjusted for in the prospective analyses.

^e^ For both the cross-sectional and prospective analyses, we adjusted for the objective ALE score in the perceived ALE score models.

| **Supplemental Table 4.** Baseline characteristics of the participants who only attended the baseline visit and participants who attended both the baseline and the follow-up visits. | | |
| --- | --- | --- |
|  | **Only baseline visit attended** | **Baseline and follow-up visits attended** |
|  | *(n=1,378)* | *(n=942)* |
|  | **mean (SD)** | |
| Age, *years* | 71.2 (7.8) | 67.6 (6.8) |
| Body mass index, *kg/m^2^* | 26.7 (4.0) | 26.3 (4.2) |
|  | **% (n)** | |
| Women | 54.4 (750) | 58.0 (546) |
| Education level |  |  |
| *O-level or lower* | 39.4 (542) | 33.9 (319) |
| *A-level* | 45.0 (619) | 46.9 (442) |
| *Degree* | 15.7 (216) | 19.2 (181) |
| Paid job at present | 17.8 (245) | 28.7 (270) |
| Married/living with partner (*vs.* single/widowed/separated/divorced) | 78.3 (1,079) | 84.9 (800) |
| Employment-based social class | 97.1 (1,338) |  |
| *Unskilled/semi-skilled* |  | 10.5 (99) |
| *Skilled (non-manual and manual)* | 12.9 (178) | 36.5 (344) |
| *Professional/managerial* | 37.6 (518) | 53.0 (499) |
| Born in the UK | 49.5 (682) | 97.5 (918) |
| Smoking status (current *vs.* former/never) | 3.8 (52) | 2.7 (25) |
| Physical disability that limits walking | 14.8 (204) | 9.6 (90) |
| Car primary mode of transport outside of work | 84.8 (1,169) | 90.8 (855) |
| Dog ownership | 16.5 (227) | 18.9 (178) |
| Self-rated health (good/very good/excellent self-rated health *vs.* fair/poor) | 83.2 (1,146) | 89.2 (840) |
| Urban home neighbourhood (*vs.* rural) | 58.5 (806) | 53.3 (502) |
|  | **mean (SD)** | |
| Objective ALE score^d^ | 0 (2.3) | 0 (2.4) |
| Population density, *residents/km^2^* | 2,102.5 (1,494.5) | 2,069.3 (1715.8) |
| Junction density, *junctions/hectare* | 0.2 (0.1) | 22.3 (11.7) |
| Land use mix, *HHI (0=most mixed; 10,000=least mixed)* | 2,840.9 (1,012.9) | 2,980.3 (1163.5) |
| Perceived ALE score | 67.5 (9.3) | 67.8 (9.3) |
|  |  |  |
| Activity at baseline |  |  |
| ST, *min/day* | 679.4 (66.9) | 667.0 (63.3) |
| LPA, *min/day* | 100.6 (27.6) | 107.5 (26.9) |
| MVPA, *min/day* | 79.5 (37.0) | 93.7 (36.9) |
| Overall PA, *cpm* | 236.0 (114.2) | 278.0 (116.5) |

Note: SD: standard deviation; ALE: active living environment; HHI: Herfindahl-Hirschman Index; ST, sedentary time; LPA, light-intensity physical activity; MVPA, moderate-to-vigorous intensity physical activity; PA, physical activity; cpm, counts per minute. We only included participants in their respective groups if they had complete data on all of the listed variables.

| **Supplemental Table 5.** Maximally-adjusted means differences (95% confidence intervals) in activity at baseline (cross-sectional analyses) and over the follow-up period (prospective analyses) for the continuous ALE measures (n=942). | | | | |
| --- | --- | --- | --- | --- |
|  | ST *(min/day)* | LPA *(min/day)* | MVPA (*min/day)* | Overall PA (*cpm)* |
|  |  |  |  |  |
| **Cross-sectional Analyses** |  |  |  |  |
| Objective ALE score | **2.5 (0.8, 4.2)** | **-1.6 (-2.4, -0.8)** | **-2.2 (-4.6, 0.3)** | -3.8 (-11.6, 4.0) |
| Population density | 0.001 (-0.001, 0.004) | **-0.001 (-0.003, -0.0001)** | **-2.6 (-5.5, 0.2)** | -4.9 (-14.0, 4.3) |
| Junction density | **0.3 (1.2, 2.3)** | **-0.2 (-0.4, -0.1)** | -1.7 (-4.1, 0.6) | -2.6 (-10.0, 4.9) |
| Land use mix | **0.004 (0.002, 0.01)** | **-0.002 (-0.004, -0.001)** | **-2.2 (-4.1, -0.3)** | -5.4 (-11.4, 0.7) |
| Perceived ALE score | -0.1 (-0.5, 0.3) | -0.1 (-0.3, 0.1) | 1.1 (-1.0, 3.2) | **8.2 (1.1, 15.2)** |
|  |  |  |  |  |
| **Prospective Analyses** |  |  |  |  |
| Objective ALE score | **0.4 (0.1**, **0.8)** | **-0.3 (-0.5**, **-0.2)** | -0.1 (-0.3, 0.1) | -0.5 (-1.1, 0.1) |
| Population density | **0.001 (0.0001, 0.001)** | **-0.0004 (-0.001**, **-0.0001)** | -0.0002 (-0.0005, 0.0001) | -0.001 (-0.002, 0.0002) |
| Junction density | 0.04 (-0.02, 0.1) | **-0.04 (-0.1**, **-0.01)** | -0.002 (-0.04, 0.03) | -0.01 (-0.1, 0.1) |
| Land use mix | **0.001 (0.0001, 0.001)** | **-0.0005 (-0.001, -0.0002)** | -0.0002 (-0.001, 0.0001) | -0.001 (-0.002, 0.0002) |
| Perceived ALE score | 0.1 (-0.02, 0.1) | -0.03 (-0.1, 0.01) | -0.02 (-0.1, 0.03) | -0.01 (-0.2, 0.1) |

Note: ALE, active living environment; ST, sedentary time; LPA, light-intensity physical activity; MVPA, moderate-to-vigorous intensity physical activity; PA, physical activity; cpm, counts per minute. Estimates represent the differences in activity (for the cross-sectional analyses) and differences in changes in activity (for the prospective analyses) for every one-unit increment in the ALE measures as assessed at baseline. Adjusted for all of the variables included in Blocks 1-3; Bolded values represent statistically significant effect estimates. Objective ALE score (Range: -7.6 to 6.1), population density (residents/km^2^), junction density (junctions/hectare), land use mix (Range: 1,350.3 to 8,726.5), perceived ALE score (Range: 37 to 94).

| **Supplemental Table 6.** Unadjusted and partially adjusted mean differences (95% confidence intervals) in activity at baseline across quartiles of the ALE measures (n=942). | | | | | | |
| --- | --- | --- | --- | --- | --- | --- |
|  | ST (*min/day)* | | | LPA (*min/day)* | | |
|  | **Model A** | **Model B** | **Model C** | **Model A** | **Model B** | **Model C** |
| **Objective ALE score** |  |  |  |  |  |  |
| Q1 (Least dense/mixed) | REF | REF | REF | REF | REF | REF |
| Q2 | **22.2 (11.0, 33.5)** | **23.3 (11.0, 35.6)** | **20.7 (10.9, 30.6)** | **-7.4 (-12.2, -2.6)** | **-8.4 (-13.6, -3.2)** | **-8.1 (-12.8, -3.3)** |
| Q3 | **29.0 (17.7, 40.3)** | **29.8 (16.7, 43.0)** | **24.1 (13.5, 34.7)** | **-7.6 (-12.4, -2.7)** | **-10.1 (-15.6, -4.5)** | **-9.6 (-14.8, -4.5)** |
| Q4 (Most dense/mixed) | **25.2 (14.0, 36.5)** | **26.6 (12.3, 40.9)** | **25.3 (13.7, 36.9)** | **-11.4 (-16.2, -6.6)** | **-14.6 (-20.6, -8.5)** | **-12.3 (-17.9, -6.7)** |
| **Population density** |  |  |  |  |  |  |
| Q1 (Least dense) | REF | REF | REF | REF | REF | REF |
| Q2 | **19.8 (8.5, 31.1)** | **21.7 (9.7, 33.6)** | **21.0 (11.3, 30.7)** | **-8.8 (-13.6, -3.9)** | **-9.8 (-14.9, -4.8)** | **-8.3 (-13.0, -3.6)** |
| Q3 | **23.4 (12.1, 34.7)** | **29.2 (15.0, 43.4)** | **20.4 (9.0, 31.9)** | **-5.7 (-10.5, -0.9)** | **-9.6 (-15.5, -3.6)** | **-10.0 (-15.6, -4.5)** |
| Q4 (Most dense) | **29.0 (17.7, 40.3)** | **34.9 (18.7, 51.1)** | **26.0 (12.9, 39.2)** | **-11.2 (-16.0, -6.3)** | **-15.2 (-22.0, -8.4)** | **-12.5 (-18.8, -6.1)** |
| **Junction density** |  |  |  |  |  |  |
| Q1 (Least dense) | REF | REF | REF | REF | REF | REF |
| Q2 | **20.7 (9.4, 32.0)** | **21.3 (9.5, 33.1)** | **16.3 (6.9, 25.7)** | **-6.2 (-11.0, -1.4)** | **-7.6 (-12.5, -2.6)** | **-7.6 (-12.1, -3.0)** |
| Q3 | **27.8 (16.5, 39.1)** | **23.8 (10.8, 36.8)** | **23.0 (12.6, 33.5)** | **-10.7 (-15.5, -5.9)** | **-11.3 (-16.7, -5.8)** | **-9.8 (-14.9, -4.8)** |
| Q4 (Most dense) | **20.1 (8.8, 31.5)** | **18.7 (5.2, 32.1)** | **19.1 (8.2, 30.0)** | **-10.0 (-14.8 -5.1)** | **-12.7 (-18.3, -7.0)** | **-10.5 (-15.7, -5.2)** |
| **Land use mix** |  |  |  |  |  |  |
| Q1 (Least mixed) | REF | REF | REF | REF | REF | REF |
| Q2 | **13.5 (2.1, 24.9)** | **11.8 (0.4, 23.3)** | 8.0 (-1.1, 17.1) | -3.6 (-8.5, 1.2) | -4.1 (-8.9, 0.7) | -3.7 (-8.1, 0.7) |
| Q3 | **13.3 (1.9, 24.7)** | 9.2 (-2.4, 20.8) | 8.3 (-1.1, 17.6) | **-4.9 (-9.8, -0.1)** | -4.0 (-8.9, 0.9) | -3.5 (-8.0, 1.0) |
| Q4 (Most Mixed) | **22.4 (11.0, 33.7)** | **21.2 (9.9, 32.5)** | **19.8 (10.8, 28.9)** | **-9.7 (-14.6, -4.9)** | **-9.7 (-14.5, -5.0)** | **-9.1 (-13.5, -4.8)** |
| **Perceived ALE score** |  |  |  |  |  |  |
| Q1 (Least activity-friendly) | REF | REF | REF | REF | REF | REF |
| Q2 | 8.4 (-3.0, 19.8) | 1.3 (-10.4, 12.9) | -1.9 (-11.1, 7.4) | -4.8 (-9.7, 0.002) | -1.9 (-6.8, 3.0) | -1.2 (-5.7, 3.2) |
| Q3 | **11.4 (0.01, 22.7)** | 0.3 (-12.2, 12.7) | -2.6 (-12.5, 7.3) | **-7.2 (-12.0, -2.3)** | -2.8 (-8.1, 2.4) | -1.3 (-6.0, 3.5) |
| Q4 (Most activity-friendly) | 7.4 (-4.2, 18.9) | -4.9 (-18.0, 8.3) | -9.5 (-20.0, 0.9) | -4.9 (-9.8, 0.01) | 0.1 (-5.4, 5.6) | 0.2 (-4.9, 5.2) |
|  | MVPA (*min/day)* | | | Overall PA *(cpm)* | | |
|  | **Model A** | **Model B** | **Model C** | **Model A** | **Model B** | **Model C** |
| **Objective ALE score** |  |  |  |  |  |  |
| Q1 (Least dense/mixed) | REF | REF | REF | REF | REF | REF |
| Q2 | **-9.2 (-15.9, -2.6)** | **-14.4 (-21.3, -7.4)** | **-12.6 (-19.3, -6.0)** | **-24.4 (-45.3, -3.6)** | **-39.5 (-61.2, -17.8)** | **-34.4 (-55.8, -13.1)** |
| Q3 | **-10.7 (-17.3, -4.0)** | **-16.8 (-24.1, -9.4)** | **-14.4 (-21.6, -7.3)** | **-34.0 (-55.0, -13.0)** | **-49.1 (-72.2, -26.0)** | **-42.1 (-65.0, -19.1)** |
| Q4 (Most dense/mixed) | -6.6 (-13.3, 0.003) | **-16.5 (-24.6, -8.5)** | **-13.0 (-20.9, -5.1)** | -7.9 (-28.9, 13.0) | **-35.8 (-61.1, -10.6)** | **-29.8 (-55.0, -4.6)** |
| **Population density** |  |  |  |  |  |  |
| Q1 (Least dense) | REF | REF | REF | REF | REF | REF |
| Q2 | **-12.4 (-19.0, -5.8)** | **-15.5 (-22.3, -8.8)** | **-12.7 (-19.3, -6.1)** | **-33.1 (-54.1, -12.1)** | **-41.5 (-62.7, -20.4)** | **-35.4 (-56.4, -14.4)** |
| Q3 | -4.5 (-11.1, 2.2) | -12.3 (-20.2, -4.3) | **-10.4 (-18.1, -2.6)** | -15.0 (-35.9, 6.0) | **-35.5 (-60.5, -10.5)** | **-28.5 (-53.3, -3.6)** |
| Q4 (Most dense) | -6.8 (-13.4, -0.2) | -16.2 (-25.3, -7.1) | **-13.6 (-22.5, -4.7)** | -10.5 (-31.5, 10.5) | **-35.8 (-64.4, -7.2)** | **-30.8 (-59.4, -2.3)** |
| **Junction density** |  |  |  |  |  |  |
| Q1 (Least dense) | REF | REF | REF | REF | REF | REF |
| Q2 | -5.7 (-12.4, 0.9) | **-10.0 (-16.7, -3.4)** | **-8.7 (-15.1, -2.3)** | -18.8 (-39.8, 2.2) | **-31.6 (-52.4, -10.8)** | **-26.2 (-46.7, -5.8)** |
| Q3 | **-12.1 (-18.7, -5.4)** | **-15.9 (-23.2, -8.7)** | **-13.2 (-20.3, -6.1)** | **-31.7 (-52.6, -10.8)** | **-41.5 (-64.3, -18.6)** | **-35.3 (-58.0, -12.6)** |
| Q4 (Most dense) | -3.8 (-10.5, 2.8) | **-12.2 (-19.8, -4.7)** | **-8.7 (-16.1, -1.3)** | -1.1 (-22.1, 19.8) | **-25.3 (-49.0, -1.6)** | -18.9 (-42.6, 4.7) |
| **Land use mix** |  |  |  |  |  |  |
| Q1 (Least mixed) | REF | REF | REF | REF | REF | REF |
| Q2 | -4.9 (-11.5, 1.8) | -4.8 (-11.2, 1.7) | -4.3 (-10.4, 1.9) | -16.0 (-37.0, 5.0) | -13.6 (-33.8, 6.5) | -12.1 (-31.8, 7.7) |
| Q3 | -5.6 (-12.3, 1.0) | -6.5 (-13.0, 0.1) | -4.7 (-11.0, 1.6) | -11.5 (-32.5, 9.5) | -14.4 (-34.9, 6.1) | -10.3 (-30.5, 9.9) |
| Q4 (Most Mixed) | **-11.9 (-18.5, -5.3)** | **-12.6 (-18.9, -6.2)** | **-10.7 (-16.8, -4.6)** | **-32.8 (-53.8, -11.8)** | **-34.5 (-54.4, -14.6)** | **-29.8 (-49.4, -10.3)** |
| **Perceived ALE score** |  |  |  |  |  |  |
| Q1 (Least activity-friendly) | REF | REF | REF | REF | REF | REF |
| Q2 | 2.5 (-4.2, 9.1) | 3.8 (-2.7, 10.4) | 3.1 (-3.2,9.4) | 17.8 (-2.9, 38.6) | 19.5 (-1.0, 40.0) | 16.7 (-3.4, 36.8) |
| Q3 | 2.7 (-3.9, 9.3) | 4.2 (-2.7,11.2) | 3.9 (-2.8,10.6) | 20.7 (-0.1, 41.4) | 20.6 (-1.2, 42.5) | 17.9 (-3.6, 39.3) |
| Q4 (Most activity-friendly) | **9.5 (2.8, 16.2)** | **10.4 (3.0, 17.7)** | **9.3 (2.3, 16.4)** | **46.5 (25.4, 67.5)** | **43.6 (20.5, 66.7)** | **40.5 (17.9, 63.1)** |

Note: ALE, active living environment; ST, sedentary time; LPA, light-intensity physical activity; MVPA, moderate-to-vigorous intensity physical activity; PA, physical activity; cpm, counts per minute; REF, reference. Bolded values represent statistically significant effect estimates. Model A: Unadjusted; Model B: Adjusted for Block 1 variables; Model C: Adjusted for Block 1& 2 variables; Quartile cutoffs: Objective ALE score (n): Q1: <-1.3 (236), Q2: ≥-1.3<0.3 (238), Q3: ≥0.3<1.6 (233), Q4: ≥1.6 (235); Population density, residents/km^2^ (n): Q1: <513.2 (236), Q2: ≥513.2<2,048.0 (235), Q3: ≥2,048.0<3,227.0 (237), Q4: ≥3,227.0 (234); Junction density, junctions/ hectare (n): Q1: <12.8 (236), Q2: ≥12.8<22.1 (235), Q3: ≥22.1<29.6 (237), Q4: ≥29.6 (234); Land use mix (n): Q1: <6,467.6 (236), Q2: ≥6,467.6<7,284.7 (235), Q3: ≥7,284.7<7,847.6 (236), Q4: ≥7,847.6 (235); Perceived ALE score (n): Q1: <62 (239), Q2: ≥62<68 (238), Q3: ≥68<74(240), Q4: ≥74 (225).

| **Supplemental Table 7.** Maximally-adjusted means differences (95% confidence intervals) in activity at baseline across quartiles of the ALE measures using the full available EPIC-Norfolk baseline dataset (i.e. 3^rd^ Health Check; n=2,626). | | | | |
| --- | --- | --- | --- | --- |
|  | ST (*min/day)* | LPA (*min/day)* | MVPA (*min/day)* | Overall PA (*cpm)* |
| **Objective ALE score** |  |  |  |  |
| Q1 (Least dense/mixed) | REF | REF | REF | REF |
| Q2 | **12.5 (6.8, 18.1)** | **-5.1 (-7.9, -2.2)** | **-7.4 (-11.1, -3.7)** | **-21.1 (-32.8, -9.3)** |
| Q3 | **15.0 (9.1, 21.0)** | **-5.4 (-8.4, -2.4)** | **-9.6 (-13.5, -5.7)** | **-27.4 (-39.7, -15.1)** |
| Q4 (Most dense/mixed) | **15.5 (9.2, 21.9)** | **-8.2 (-11.4, -5.0)** | **-7.4 (-11.5, -3.2)** | **-16.2 (-29.4, -3.0)** |
| **Population density** |  |  |  |  |
| Q1 (Least dense) | REF | REF | REF | REF |
| Q2 | **12.2 (6.7, 17.7)** | **-4.3 (-7.1, -1.6)** | **-7.9 (-11.5, -4.3)** | **-21.6 (-33.0, -10.2)** |
| Q3 | **16.0 (9.6, 22.3)** | **-6.3 (-9.5, -3.1)** | **-9.7 (-13.8, -5.5)** | **-25.8 (-39.0, -12.7)** |
| Q4 (Most dense) | **18.4 (11.2, 25.5)** | **-7.4 (-11.0, -3.8)** | **-11.0 (-15.6, -6.3)** | **-28.7 (-43.5, -13.9)** |
| **Junction density** |  |  |  |  |
| Q1 (Least dense) | REF | REF | REF | REF |
| Q2 | **12.2 (6.6, 17.8)** | **-4.9 (-7.7, -2.1)** | **-7.3 (-11.0, -3.6)** | **-21.2 (-32.8, -9.6)** |
| Q3 | **15.1 (9.3, 20.8)** | **-6.1 (-9.0, -3.2)** | **-9.0 (-12.7, -5.2)** | **-25.4 (-37.4, -13.5)** |
| Q4 (Most dense) | **13.2 (7.2, 19.1)** | **-6.7 (-9.7, -3.7)** | **-6.5 (-10.4, -2.6)** | **-15.4 (-27.7, -3.1)** |
| **Land use mix** |  |  |  |  |
| Q1 (Least mixed) | REF | REF | REF | REF |
| Q2 | **6.5 (1.3, 11.7)** | **-2.6 (-5.2, -0.02)** | **-3.9 (-7.3, -0.5)** | **-11.0 (-21.8, -0.2)** |
| Q3 | 3.7 (-1.5, 9.0) | -2.0 (-4.7, 0.6) | -1.7 (-5.1, 1.8) | -0.3 (-11.2, 10.6) |
| Q4 (Most Mixed) | **9.4 (4.2, 14.6)** | **-5.4 (-8.1, -2.8)** | **-4.0 (-7.4, -0.5)** | -6.5 (-17.3, 4.3) |
| **Perceived ALE score** |  |  |  |  |
| Q1 (Least activity-friendly) | REF | REF | REF | REF |
| Q2 | -0.9 (-6.1, 4.4) | 0.6 (-2.1, 3.2) | 0.3 (-3.2, 3.7) | 2.4 (-8.6, 13.3) |
| Q3 | -3.6 (-8.9, 1.8) | 1.0 (-1.7, 3.7) | 2.6 (-0.9, 6.1) | 7.0 (-4.2, 18.1) |
| Q4 (Most activity-friendly) | -3.5 (-9.2, 2.3) | 1.9 (-0.9, 4.8) | 1.5 (-2.2, 5.3) | 6.3 (-5.6, 18.3) |

Note: ALE, active living environment; ST, sedentary time; LPA, light-intensity physical activity; MVPA, moderate-to-vigorous intensity physical activity; PA, physical activity; cpm, counts per minute; REF, reference. Bolded values represent statistically significant effect estimates. Adjusted for all of the variables included in Blocks 1-3; Quartile cutoffs: Objective ALE score (n): Q1: <-1.0 (657), Q2: ≥-1.0<0.2 (656), Q3: ≥0.2<1.5 (657), Q4: ≥1.5 (656); Population density, residents/km^2^ (n): Q1: <648.5 (657), Q2: ≥648.5< 2,115.5 (656), Q3: ≥2,115.5<3,182.6 (658), Q4: ≥3,182.6 (655); Junction density, junctions/hectare (n): Q1: <15.4 (657), Q2: ≥15.4<23.1 (658), Q3: ≥23.1<31.1 (655), Q4: ≥31.1 (656); Land use mix (n): Q1: <6,646.1 (657), Q2: ≥6,646.1<7,369.2 (657), Q3: ≥7,369.2<7,874.2 (656), Q4: ≥7,874.2 (656); Perceived ALE score (n): Q1: <62 (703), Q2: ≥62<68 (624), Q3: ≥68<74(693), Q4≥74 (606).

| **Supplemental Table 8.** Unadjusted and partially adjusted mean differences (95% confidence intervals) in rates of change in activity between the baseline and follow-up visits across quartiles of the ALE measures (n=942). | | | | | | |
| --- | --- | --- | --- | --- | --- | --- |
|  | ST (*min/day/year)* | | | LPA (*min/day/year)* | | |
|  | **Model A** | **Model B** | **Model C** | **Model A** | **Model B** | **Model C** |
| **Objective ALE score** |  |  |  |  |  |  |
| Q1 (Least dense/mixed) | REF | REF | REF | REF | REF | REF |
| Q2 | **-2.3 (-4.6, -0.1)** | **-2.6 (-5.0, -0.1)** | 0.4 (-1.4, 2.2) | 0.4 (-0.5, 1.4) | 0.2 (-0.8, 1.2) | -0.4 (-1.3, 0.4) |
| Q3 | **-3.0 (-5.3, -0.7)** | **-3.2 (-5.8, -0.5)** | 1.1 (-0.9, 3.1) | 0.03 (-0.9, 1.0) | -0.1 (-1.2, 1.0) | -0.9 (-1.8, 0.1) |
| Q4 (Most dense/mixed) | -0.7 (-2.9, 1.6) | -0.7 (-3.6, 2.2) | **2.4 (0.2, 4.6)** | 0.4 (-0.6, 1.3) | 0.1 (-1.1, 1.4) | **-1.4 (-2.5, -0.4)** |
| **Population density** |  |  |  |  |  |  |
| Q1 (Least dense) | REF | REF | REF | REF | REF | REF |
| Q2 | -2.0 (-4.3, 0.3) | -2.2 (-4.6, 0.3) | 0.9 (-0.9, 2.7) | 0.6 (-0.4, 1.5) | 0.4 (-0.6, 1.4) | -0.4 (-1.3, 0.4) |
| Q3 | -2.0 (-4.3, 0.2) | -2.3 (-5.2, 0.5) | 1.9 (-0.3, 4.0) | 0.3 (-0.6, 1.3) | -0.02 (-1.2, 1.2) | -0.8 (-1.9, 0.2) |
| Q4 (Most dense) | -1.2 (-3.5, 1.0) | -1.5 (-4.8, 1.8) | 2.1 (-0.3, 4.6) | 0.2 (-0.7, 1.2) | -0.3 (-1.7, 1.1) | **-1.6 (-2.8, -0.4)** |
| **Junction density** |  |  |  |  |  |  |
| Q1 (Least dense) | REF | REF | REF | REF | REF | REF |
| Q2 | -1.2 (-3.4, 1.1) | -1.3 (-3.7, 1.0) | 1.4 (-0.4, 3.1) | -0.2 (-1.2, 0.7) | -0.4 (-1.4, 0.6) | **-0.9 (-1.7, -0.01)** |
| Q3 | **-2.7 (-5.0, -0.5)** | **-2.8 (-5.4, -0.1)** | 1.3 (-0.6, 3.3) | -0.2 (-1.1, 0.8) | -0.4 (-1.4, 0.7) | **-1.2 (-2.1, -0.2)** |
| Q4 (Most dense) | -0.4 (-2.7, 1.9) | -0.1 (-2.8, 2.6) | **2.5 (0.5, 4.6)** | 0.4 (-0.5, 1.4) | 0.2 (-1.0, 1.3) | **-1.2 (-2.2, -0.2)** |
| **Land use mix** |  |  |  |  |  |  |
| Q1 (Least mixed) | REF | REF | REF | REF | REF | REF |
| Q2 | 1.3 (-1.0, 3.6) | 1.2 (-1.1, 3.5) | **2.0 (0.3, 3.6)** | -0.6 (-1.5, 0.4) | -0.5 (-1.5, 0.4) | **-1.0 (-1.8, -0.2)** |
| Q3 | -0.4 (-2.6, 1.9) | -0.4 (-2.7, 2.0) | 1.4 (-0.3, 3.1) | 0.0002 (-0.9, 0.9) | -0.1 (-1.1, 0.9) | -0.5 (-1.4, 0.3) |
| Q4 (Most Mixed) | -0.8 (-3.0, 1.5) | -0.9 (-3.2, 1.4) | 1.3 (-0.4, 3.0) | -0.2 (-1.2, 0.7) | -0.2 (-1.2, 0.7) | **-1.2 (-2.0, -0.3)** |
| **Perceived ALE score** |  |  |  |  |  |  |
| Low | REF | REF | REF | REF | REF | REF |
| Low-middle | 0.9 (-1.3, 3.2) | 1.4 (-0.9, 3.8) | 0.1 (-1.6, 1.8) | -0.1 (-1.1, 0.8) | -0.1 (-1.0, 0.9) | -0.4 (-1.3, 0.4) |
| Middle-high | 0.9 (-1.4, 3.2) | 1.8 (-0.7, 4.4) | 0.2 (-1.6, 2.0) | 0.6 (-0.4, 1.5) | 0.5 (-0.5, 1.6) | 0.1 (-0.8, 1.0) |
| High | 0.4 (-1.9, 2.7) | 1.6 (-1.0, 4.3) | -0.3 (-2.2, 1.6) | 0.5 (-0.5, 1.4) | 0.3 (-0.8, 1.4) | -0.1 (-1.0, 0.8) |
|  | MVPA (*min/day/year)* | | | Overall PA *(cpm/year)* | | |
|  | **Model A** | **Model B** | **Model C** | **Model A** | **Model B** | **Model C** |
| **Objective ALE score** |  |  |  |  |  |  |
| Q1 (Least dense/mixed) | REF | REF | REF | REF | REF | REF |
| Q2 | **1.5 (0.4, 2.6)** | **1.5 (0.3, 2.7)** | 0.3 (-0.7, 1.4) | **3.7 (0.3, 7.2)** | **4.1 (0.3, 7.9)** | 0.5 (-2.9, 3.9) |
| Q3 | 1.0 (-0.1, 2.1) | 1.2 (-0.1, 2.4) | -0.3 (-1.4, 0.8) | **3.7 (0.2, 7.2)** | 4.1 (-0.01, 8.2) | -0.6 (-4.3, 3.1) |
| Q4 (Most dense/mixed) | 1.0 (-0.1, 2.1) | 1.1 (-0.3, 2.5) | -0.7 (-1.9, 0.5) | 1.3 (-2.1, 4.8) | 1.7 (-2.8, 6.1) | -2.4 (-6.5, 1.6) |
| **Population density** |  |  |  |  |  |  |
| Q1 (Least dense) | REF | REF | REF | REF | REF | REF |
| Q2 | 1.4 (0.3, 2.5) | 1.5 (0.3, 2.7) | 0.1 (-0.9, 1.2) | 3.1 (-0.4, 6.6) | 3.5 (-0.3, 7.2) | -0.5 (-3.8, 2.9) |
| Q3 | 1.0 (-0.1, 2.0) | 1.1 (-0.3, 2.4) | -0.1 (-1.3, 1.1) | 3.1 (-0.3, 6.6) | 3.6 (-0.8, 8.1) | -0.1 (-4.1, 3.8) |
| Q4 (Most dense) | 1.1 (0.01, 2.2) | 1.2 (-0.3, 2.8) | -0.4 (-1.8, 1.0) | 1.9 (-1.6, 5.4) | 2.6 (-2.4, 7.7) | -1.4 (-5.9, 3.2) |
| **Junction density** |  |  |  |  |  |  |
| Q1 (Least dense) | REF | REF | REF | REF | REF | REF |
| Q2 | 0.3 (-0.7, 1.4) | 0.2 (-0.9, 1.4) | -0.5 (-1.5, 0.5) | 1.1 (-2.4, 4.6) | 0.9 (-2.8, 4.5) | -1.8 (-5.0, 1.5) |
| Q3 | 1.0 (-0.1, 2.1) | 1.0 (-0.2, 2.3) | -0.3 (-1.4, 0.8) | 3.1 (-0.4, 6.5) | 3.2 (-0.8, 7.3) | -0.7 (-4.3, 2.9) |
| Q4 (Most dense) | 0.8 (-0.3, 1.8) | 0.6 (-0.8, 1.9) | -0.9 (-2.1, 0.2) | 0.6 (-2.9, 4.1) | 0.1 (-4.1, 4.3) | -3.0 (-6.8, 0.8) |
| **Land use mix** |  |  |  |  |  |  |
| Q1 (Least mixed) | REF | REF | REF | REF | REF | REF |
| Q2 | -0.02 (-1.1, 1.1) | -0.01 (-1.1, 1.1) | -0.6 (-1.5, 0.4) | 0.3 (-3.1, 3.8) | 0.3 (-3.3, 3.8) | -1.2 (-4.4, 1.9) |
| Q3 | 0.02 (-1.1, 1.1) | -0.1 (-1.3, 1.0) | -0.8 (-1.8, 0.1) | -1.3 (-4.8, 2.2) | -1.7 (-5.3, 1.9) | **-3.4 (-6.6, -0.2)** |
| Q4 (Most Mixed) | 0.6 (-0.5, 1.7) | 0.6 (-0.5, 1.7) | -0.6 (-1.5, 0.4) | 1.4 (-2.1, 4.9) | 1.3 (-2.2, 4.8) | -1.9 (-5.1, 1.2) |
| **Perceived ALE score** |  |  |  |  |  |  |
| Q1 (Least activity-friendly) | REF | REF | REF | REF | REF | REF |
| Q2 | -0.1 (-1.1, 1.0) | -0.2 (-1.3, 0.9) | -0.2 (-1.1, 0.8) | -1.5 (-5.0, 2.0) | -1.7 (-5.3, 1.9) | -0.3 (-3.5, 2.9) |
| Q3 | 0.4 (-0.7, 1.5) | -0.2 (-1.4, 1.0) | -0.1 (-1.1, 0.9) | 0.1 (-3.3, 3.6) | -0.7 (-4.6, 3.2) | 0.8 (-2.6, 4.3) |
| Q4 (Most activity-friendly) | 0.5 (-0.6, 1.6) | -0.1 (-1.4, 1.1) | 0.3 (-0.8, 1.4) | -0.1 (-3.6, 3.4) | -1.0 (-5.1, 3.1) | 2.2 (-1.4, 5.8) |

Note: ALE, active living environment; ST, sedentary time; LPA, light-intensity physical activity; MVPA, moderate-to-vigorous intensity physical activity; PA, physical activity; cpm, counts per minute; Bolded values represent statistically significant effect estimates; Model A: Unadjusted; Model B: Adjusted for Block 1 variables (age, education, employment, change in employment, marital status, change in marital status, immigrant status, social class, urban status); Model C: Model B + Block 2 variables (sex, smoking status, season of assessment, change in season of assessment, accelerometer wear-time, change in accelerometer wear-time, physical disability, baseline activity); Quartile cutoffs: Objective ALE score (n): Q1: <-1.3 (236), Q2: ≥-1.3<0.3 (238), Q3: ≥0.3<1.6 (233), Q4: ≥1.6 (235); Population density, residents/km^2^ (n): Q1: <513.2 (236), Q2: ≥513.2<2,048.0 (235), Q3: ≥2,048.0<3,227.0 (237), Q4: ≥3,227.0 (234); Junction density, junctions/hectare (n): Q1: <12.8 (236), Q2: ≥12.8<22.1 (235), Q3: ≥22.1<29.6 (237), Q4: ≥29.6 (234); Land use mix (n): Q1: <6,467.6 (236); Q2: ≥6,467.6<7,284.7 (235), Q3: ≥7,284.7<7,847.6 (236), Q4: ≥7,847.6 (235); Perceived ALE score (n): Q1: <62 (239), Q2: ≥62<68 (238), Q3: ≥68<74(240), Q4: ≥74 (225).
